# European Kidney Function Consortium Equation vs. Chronic Kidney Disease Epidemiology Collaboration (CKD-EPI) Refit Equations for Estimating Glomerular Filtration Rate: Comparison with CKD-EPI Equations in the Korean Population

**DOI:** 10.3390/jcm11154323

**Published:** 2022-07-25

**Authors:** Hanah Kim, Mina Hur, Seungho Lee, Gun-Hyuk Lee, Hee-Won Moon, Yeo-Min Yun

**Affiliations:** 1 Department of Laboratory Medicine, Konkuk University School of Medicine, Konkuk University Medical Center, 120-1, Neungdong-ro, Hwayang-dong, Gwangjin-gu, Seoul 05030, Korea; md.hkim@gmail.com (H.K.); leegunhyuk93@gmail.com (G.-H.L.); hannasis@hanmail.net (H.-W.M.); ymyun@kuh.ac.kr (Y.-M.Y.); 2 Department of Preventive Medicine, Dong-A University College of Medicine, Busan 49201, Korea; lgydr1@gmail.com

**Keywords:** glomerular filtration rate, equation, CKD-EPI, EKFC, CKD-EPI refit, comparison

## Abstract

The Chronic Kidney Disease Epidemiology Collaboration (CKD-EPI) equation is the most commonly used equation for estimated glomerular filtration rate (eGFR). Recently, the European Kidney Function Consortium (EKFC) announced a full-age spectrum equation, and the CKD-EPI announced the CKD-EPI refit equations (CKD-EPI-R). We compared CKD-EPI, EKFC, and CKD-EPI-R equations in a large-scale Korean population and investigated their potential implications for CKD prevalence. In a total of 106,021 individuals who received annual check-ups from 2018 to 2020, we compared the eGFR equations according to the Clinical and Laboratory Standards Institute guidelines. Weighted kappa (κ) agreement was used to compare the potential implications for CKD prevalence across the equations. The median value of eGFR tended to increase in the order of EKFC, CKD-EPI, and CKD-EPI-R equations (92.4 mL/min/1.73 m^2^, 96.0 mL/min/1.73 m^2^, and 100.0 mL/min/1.73 m^2^, respectively). The EKFC and CKD-EPI-R equations showed a very high correlation of eGFR and good agreement for CKD prevalence with CKD-EPI equation (r = 0.98 and 1.00; κ = 0.80 and 0.82, respectively). Compared with the CKD-EPI equation, the EFKC equation overestimated CKD prevalence (3.5%), and the CKD-EPI-R equation underestimated it (1.5%). This is the first study comparing CKD-EPI, EKFC, and CKD-EPI-R equations simultaneously. The EKFC and CKD-EPI-R equations were statistically interchangeable with CKD-EPI equations in this large-scale Korean population. The transition of eGFR equations, however, would lead to sizable changes in the CKD prevalence. To improve kidney health, in-depth discussion considering various clinical aspects is imperative for the transition of eGFR equations.

## 1. Introduction

In the 2019 Global Burden of Disease Study, chronic kidney disease (CKD) is ranked as the 18th cause of disability-adjusted life years and 11th cause of deaths [1]. The prevalence of CKD is approximately 8.6% worldwide and 9.3% in the Korean population [1,2]. Glomerular filtration rate (GFR) is the most valuable single index for assessing kidney function and is used for diagnosis and staging of CKD according to the Kidney Disease Improving Global Outcomes (KDIGO) guidelines [3]. Direct measurement of GFR using inulin clearance or radio-isotope is, however, impractical in clinical practice; therefore, estimated GFR (eGFR) using serum levels of endogenous filtration markers, such as creatinine (sCr) and cystatin C (sCysC), is commonly used [4].

The CKD prevalence can be affected by eGFR equations. Many GFR equations have been developed, and the most commonly used eGFR equations are the Chronic Kidney Disease Epidemiology Collaboration (CKD-EPI) equation for adults and the Chronic Kidney Disease in Children Study (CKiD) equation for children [3,4,5]. These equations, however, have limitations in patients at the transition age from adolescent to adult (18–39 years (yrs)). First, CKD-EPI and CKiD equations overestimate eGFR [6,7,8]. Second, consecutive eGFRs might show significant differences despite no change in sCr levels when a patient transitions from adolescence to young adulthood [5,6,7,8]. In 2020, the European Kidney Function Consortium (EKFC) announced a new full-age spectrum eGFR equation that can be used across the entire age range. The EKFC equation was developed and validated using European and US cohorts [6,7,8,9,10,11,12,13,14,15]; in the Asian populations, it was evaluated only in a few studies [16,17].

In 2021, the CKD-EPI announced new sCr- and sCysC-based eGFR refit equations (CKD-EPI-R) that omitted race, because race is a social, and not a biological, construct [18]. They reported that the new CKD-EPI-R equation underestimated measured GFR (mGFR) in Blacks and overestimated it in non-Blacks. Therefore, it would increase the CKD prevalence among Blacks and yield similar or lower CKD prevalence among non-Blacks [18,19,20,21]. However, the Chronic Renal Insufficiency Cohort (CRIC) Study noticed that eliminating race from eGFR equations alone may not significantly affect the end-stage kidney disease (ESKD) prediction and kidney health inequities [22,23]. Furthermore, the CKD-EPI-R equations were developed and validated using only the European and US cohorts that consisted of a very small number of Asians [18].

Even though the newly proposed eGFR equations can be applied globally, their evaluation is essential before being adopted in each region other than North America, Europe, and Australia [3,4,20]. The CKD-EPI equations have been evaluated and compared with mGFR or other eGFR equations in several studies using the Korean populations, and most of these comparisons have focused on sCr-based eGFRs only [24,25,26,27,28,29,30,31,32]. Furthermore, there is no study that evaluated the newly proposed EKFC and CKD-EPI-R equations compared with the CKD-EPI equations in the Asian population.

In this study, we aimed to compare eGFRs, including sCysC-based eGFRs, using the newly proposed EKFC and CKD-EPI-R equations in comparison with the CKD-EPI equations. We compared these equations using the approved statistical analyses according to the Clinical and Laboratory Standards Institute (CLSI) guidelines in a large-scale Korean general population. We also investigated the potential implications for CKD prevalence across the eGFR equations.

## 2. Methods

### 2.1. Study Population

We reviewed the data collected from a total of 106,295 individuals who received annual physical check-ups at Konkuk University Medical Center (KUMC), Seoul, Korea, from January 2018 to November 2020. All individuals followed the requested instructions for preparation for physical check-ups. To reduce the influence on the levels of sCr, sCysC, and other laboratory parameters, they were asked to refrain from behaviors that may affect the testing results, such as drinking, meat, oily food, and overwork for 2 to 3 days before check-ups. In addition, they were asked to keep fasting at least 8 h before the check-ups. Each individual declared his or her nationality as a Korean. After excluding 274 individuals who were aged less than 20 yrs and/or missing data for age, gender, and sCr levels, we included 106,021 individuals (51,503 males (48.6%); median age of 48 yrs, inter-quartile range (IQR) 39–58 yrs). Their median level of sCr was 0.82 mg/dL (IQR, 0.70–0.96 mg/dL). All individuals confirmed that they had not received renal replacement therapy or a kidney transplant, based on their self-report. The sCr-based eGFRs were compared in all individuals, and sCr- and sCysC-based eGFRs were compared in 12,635 individuals with both sCr and sCysC level data (6248 males (49.4%); median age of 50 yrs, IQR 40–59 yrs). Their median level of sCr was 0.82 mg/dL (IQR, 0.69–0.96 mg/dL) and that of sCysC was 0.80 mg/dL (IQR, 0.70–0.91 mg/dL).

This study was approved by the Institutional Review Board of KUMC (IRB number: KUMC 2021-04-007). This study was conducted using anonymously collected age, gender, sCr level, and sCysC level from the enrolled individuals, without any clinical manipulations; accordingly, obtaining written informed consent from the enrolled individuals was waived.

### 2.2. Estimation of GFR

The sCr levels were determined by the modified Jaffe compensated method using Creatinine FS (DiaSys Diagnostic Systems GmbH, Holzheim, Germany) reagent on an automated chemistry analyzer, TBA-FX8 (Canon Medical Systems Corporation, Tokyo, Japan). Isotope-dilution mass spectrometry (IDMS)-traceable calibration was conducted daily with a TruCal U (DiaSys Diagnostic Systems GmbH) calibrator. For internal quality control of the sCr assay, two levels of liquid assayed chemistry quality control materials, the Liquid Assayed Multiqual Level 1, 3 (Bio-Rad Laboratories, Inc., Hercules, CA, USA), were tested twice a day. The mean within laboratory precision (CV) of the sCr assay was 3.66% during the study period, and the available measurable range of sCr was 0.2–25 mg/dL.

The sCysC levels were determined by the turbidimetric immunoassay using Gentian Cystatin C Immunoassay Reagent Kit (Gentian Diagnostics AS, Moss, Norway) on TBA-FX8. The calibration was conducted daily with a Gentian Cystatin C Calibrator Kit (calibrated against reference material ERM-DA471/IFCC). For internal quality control of the sCysC assay, the Gentian Cystatin C Control Kit was tested once a day. The mean within CV of the sCysC assay was 4.12% during the study period, and the available measurable range of sCysC was 0.4–8.0 mg/dL.

The laboratory participated in the external proficiency testing program organized by the Korean Association of Quality Assurance for Clinical Laboratory, and the results of sCr and sCysC measurements were all acceptable (variance index scores < 150) during the study period. The eGFR (mL/min/1.73 m^2^) was estimated using the CKD-EPI, EKFC, and CKD-EPI-R equations [1,7,14]. The GFR categories were assigned according to the KDIGO 2012 guidelines, and the potential CKD prevalence was defined as eGFR less than 60 mL/min/1.73 m^2^ [1].

### 2.3. Statistical Analysis

Data were expressed as the median and IQR for continuous variables and as numbers and percentages for categorical or binary variables. The Wilcoxon test was used for a paired sample comparison. The eGFRs using EFKC and CKD-EPI-R equations were compared with eGFR using CKD-EPI equations using the Passing–Bablok regression and the Bland–Altman plots, according to the CLSI guidelines (EP09C-ED3) [33]. In the Passing–Bablok regression, the correlation coefficients (r) were interpreted as follows: <0.30, negligible; 0.30–0.49, low; 0.50–0.69, moderate; 0.70–0.89, high; and ≥0.90, very high correlations [34]. In the Bland–Altman plots, the results were interpreted informally to observe how big the mean difference is and whether there is a trend of difference [35].

Weighted kappa (κ) value was used to calculate the agreement degree of CKD categories and CKD prevalence. The κ values were interpreted as follows: <0.20, poor; 0.21–0.40, fair; 0.41–0.60, moderate; 0.61–0.80, good; and >0.81, very good [36]. The rate difference and 95% confidence interval (CI) of potential CKD prevalence were calculated using the test-based method [37]. The rate ratio and 95% CI of potential CKD prevalence were calculated using the exact Poisson method [38]. Statistical analyses were conducted using the MedCalc Statistical Software (version 20.015, MedCalc Software Ltd., Ostend, Belgium), and a two-tailed *p* value less than 0.05 was considered statistically significant.

## 3. Results

### 3.1. eGFR Based on sCr Level

**Figure 1:**
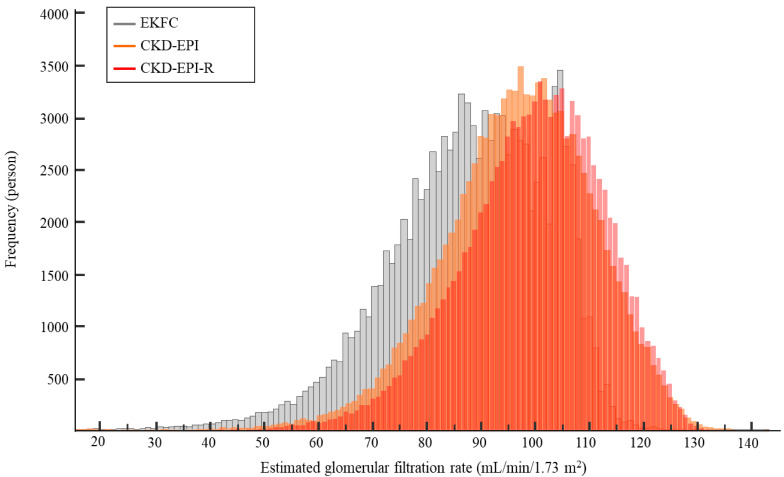
Distribution of eGFR using serum creatinine-based CKD-EPI, EKFC, and CKD-EPI-R equations. Abbreviations: see Table 1.

**Figure 2:**
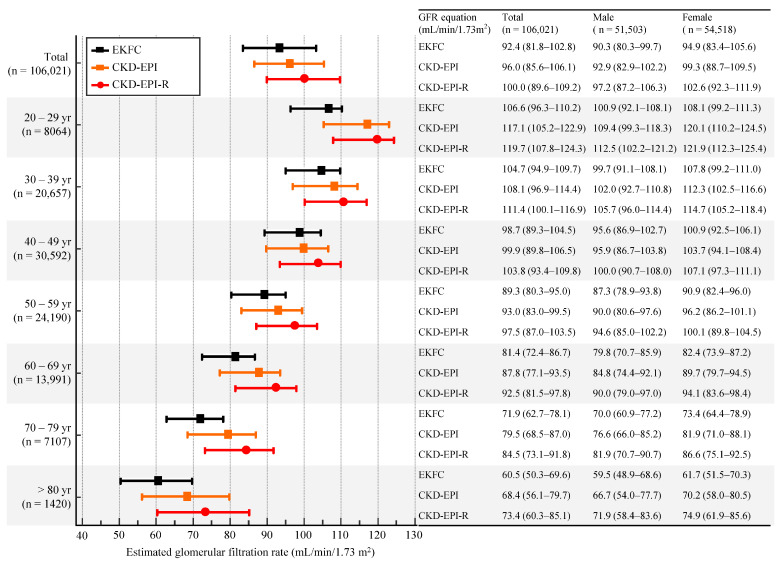
Distribution of eGFR using serum creatinine-based CKD-EPI, EKFC, and CKD-EPI-R equations stratified by age group and gender. Data are presented as median and interquartile range. Abbreviations: see Table 1.

Figure 1 shows the distribution of eGFR using CKD-EPI, EKFC, and CKD-EPI-R equations; the median value of eGFRs tended to increase from 92.4 mL/min/1.73 m^2^ using EKFC to 96.0 mL/min/1.73 m^2^ using CKD-EPI to 100.0 mL/min/1.73 m^2^ using CKD-EPI-R equation. When the eGFR distribution was stratified by age group, such a trend was consistently observed throughout the entire age group (Figure 2). Overall, the EKFC equation showed the lowest eGFRs, followed by CKD-EPI and CKD-EPI-R equations, regardless of age and gender. At the transition age groups (20–29 yrs and 30–39 yrs), the EKFC equation showed the smallest differences of eGFRs between the two groups of 20s and 30s, compared with CKD-EPI and CKD-EPI-R equations (1.9 mL/min/1.73 m^2^ vs. 9.0 mL/min/1.73 m^2^ vs. 8.3 mL/min/1.73 m^2^).

**Figure 3:**
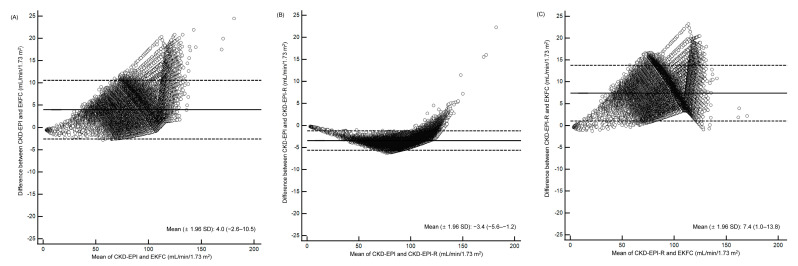

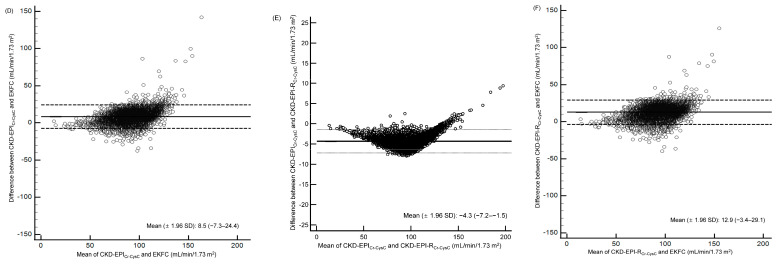
Bland–Altman plots to compare CKD-EPI, EKFC, and CKD-EPI-R equations. (**A**–**C**) Differences of eGFR among serum creatinine-based CKD-EPI, EKFC, and CKD-EPI-R equations (n = 106,021). (**D**–**F**) Differences of eGFR among cystatin C-based CKD-EPI, EKFC, and CKD-EPI-R equations (n = 12,635). Solid lines indicate mean difference, and dashed lines indicate ±1.96 standard deviations. Abbreviations: see Table 3.

Table 1 and Figure 3 shows correlation and mean difference of eGFRs across CKD-EPI, EKFC, and CKD-EPI-R equations using the Passing–Bablok regression and the Bland–Altman plot. Both EKFC and CKD-EPI-R equations showed a very high correlation with CKD-EPI equation (r = 0.98 and 1.00, respectively) in all age groups. The mean differences of eGFRs across the equations were: 4.0 mL/min/1.73 m^2^ for CKD-EPI vs. EKFC; −3.4 mL/min/1.73 m^2^ for CKD-EPI vs. CKD-EPI-R; 7.4 mL/min/1.73 m^2^ for CKD-EPI-R vs. EKFC. The EKFC and CKD-EPI-R equations, however, showed greater than 10% of mean differences in young and old age groups.

The EKFC and CKD-EPI-R equations showed good to very good agreement (κ = 0.80 and 0.82, respectively) to the CKD-EPI equation for potential CKD prevalence (Table 2). The estimated CKD prevalence was 2.1% (2202/106,021) using the CKD-EPI equation. Using the EKFC equation, the estimated CKD prevalence was 3.0% (3221/106,021); 1031 of 106,021 individuals (1.0%) were reclassified as having CKD, while 12 of 106,021 were reclassified as not having CKD. Using the CKD-EPI-R equation, the estimated CKD prevalence was 1.5% (1540/106,021); none of the enrolled population was reclassified as having CKD, while 662 of 106,021 individuals (0.6%) were reclassified as not having CKD.

**Table 1:** Correlation and differences of eGFR among serum creatinine-based CKD-EPI, EKFC, and CKD-EPI-R equations stratified by age group.

Age (yr)	CKD−EPI vs. EKFC			CKD−EPI vs. CKD−EPI−R			CKD−EPI−R vs. EKFC		
	Equation	r (95% CI)	Mean Difference (95% CI)	Equation	r (95% CI)	Mean Difference (95% CI)	Equation	r (95% CI)	Mean Difference (95% CI)
20–29 (n = 8064)	y = 0.81x + 11.59	0.97 (0.97–0.97)	10.6 (4.6–16.6)	y = 0.93x + 9.38	1.00 (1.00–1.00)	−1.9 (−4.1–0.4)	y = 0.86x + 4.37	0.97 (0.97–0.97)	12.5 (7.6–17.4)
30–39 (n = 20,657)	y = 0.86x + 10.98	0.97 (0.97–0.97)	3.5 (−1.7–8.6)	y = 0.95x + 7.81	1.00 (1.00–1.00)	−2.7 (−4.7–−0.7)	y = 0.91x + 3.93	0.97 (0.97–0.97)	6.1 (1.8–10.4)
40–49 (n = 30,592)	y = 0.93x + 5.65	0.99 (0.99–0.99)	1.1 (−1.8–4.1)	y = 0.97x + 6.26	1.00 (1.00–1.00)	−3.4 (−5.1–−1.7)	y = 0.96x–0.92	0.99 (0.99–0.99)	4.5 (1.8–7.2)
50–59 (n = 24,190)	y = 0.90x + 5.13	0.99 (0.99–0.99)	3.6 (0.4–6.7)	y = 0.99x + 5.22	1.00 (1.00–1.00)	−3.9 (−5.4–−2.3)	y = 0.92x–0.01	1.00 (0.99–1.00)	7.4 (4.4–10.5)
60–69 (n = 13,991)	y = 0.88x + 4.21	0.99 (0.99–0.99)	5.8 (2.3–9.3)	y = 0.99x + 4.82	1.00 (1.00–1.00)	−4.2 (−5.6–−2.7)	y = 0.89x–0.30	1.00 (1.00–1.00)	10.0 (6.4–13.6)
70–79 (n = 7107)	y = 0.86x + 3.78	1.00 (1.00–1.00)	7.2 (3.0–11.4)	y = 1.01x + 3.42	1.00 (1.00–1.00)	−4.4 (−6.0–−2.9)	y = 0.85x + 0.81	1.00 (1.00–1.00)	11.6 (6.9–16.4)
>80 (n = 1420)	y = 0.84x + 3.17	1.00 (1.00–1.00)	7.6 (2.5–12.6)	y = 1.04x + 1.70	1.00 (1.00–1.00)	−4.5 (−6.3–−2.8)	y = 0.81x + 1.57	1.00 (1.00–1.00)	12.1 (5.8–18.4)
Total (n = 106,021)	y = 0.94x + 2.12	0.98 (0.98–0.98)	4.0 (−2.6–10.5)	y = 0.96x + 7.10	1.00 (1.00–1.00)	−3.4 (−5.6–−1.2)	y = 0.97x–4.57	0.98 (0.97–0.99)	7.4 (1.0–13.8)

All *p* values were <0.001. Equations and correlation coefficients (r) were obtained using Passing–Bablok regression, and mean differences were obtained using Bland–Altman plots.

**Table 2:** Agreement of GFR categories among serum creatinine-based CKD-EPI, EKFC, and CKD-EPI-R equations.

	GFR Category	CKD-EPI							Categorical Agreement		Agreement of Estimated CKD Prevalence			
		G1	G2	G3a	G3b	G4	G5	Total	%	κ (95% CI)	κ (95% CI)	Estimated CKD Prevalence (%)	Difference and Rate in Prevalence (%, 95% CI)	*p*
EKFC	G1	59,439	291	0	0	0	0	59,730	89.1	0.80 (0.80–0.81)	0.80 (0.79–0.81)	3.0	1.0 (0.8–1.1) 1.5 (1.4–1.5)	< 0.001
	G2	9954	33,104	12	0	0	0	43,070						
	G3a	0	1031	1461	1	0	0	2493						
	G3b	0	0	194	349	4	0	547						
	G4	0	0	0	13	80	4	97						
	G5	0	0	0	0	0	84	84						
CKD-EPI-R	G1	69,393	9346	0	0	0	0	78,739	90.4	0.80 (0.79–0.80)	0.82 (0.81–0.83)	1.5	−0.6 (−0.7–−0.5) 0.7 (0.7–0.7)	< 0.001
	G2	0	25,080	662	0	0	0	25,742						
	G3a	0	0	1005	120	0	0	1125						
	G3b	0	0	0	243	20	0	263						
	G4	0	0	0	0	64	4	68						
	G5	0	0	0	0	0	84	84						
Total		69,393	34,426	1667	363	84	88	106,021						

Estimated CKD prevalence using CKD-EPI equation was 2.1%. Abbreviations: CI, confidence interval; CKD-EPI, Chronic Kidney Disease Epidemiology Collaboration; CKD-EPI-R, Chronic Kidney Disease Epidemiology Collaboration refit; EKFC, The European Kidney Function Consortium; eGFR, estimated glomerular filtration rate; r, correlation coefficient; yr, years.

**Table 3:** Distribution of eGFR using serum creatinine- and cystatin C-based CKD-EPI, EKFC, and CKD-EPI-R equations stratified by age group.

	20–29 yr (n *=* 541)	30–39 yr (n = 2382)	40–49 yr (n = 3271)	50–59 yr (n = 3447)	60–69 yr (n = 2217)	70–79 yr (n = 702)	>80 yr (n = 75)	Total (n = 12,635)
CKD-EPI_Cr-CysC_	106.7 (96.3–115.7)	112.9 (103.3–121.0)	106.0 (96.4–113.9)	96.2 (86.2–104.5)	88.3 (77.8–97.8)	78.9 (67.1–88.3)	69.3 (54.5–80.3)	100.4 (88.0–111.0)
EKFC	105.0 (95.2–110.0)	105.6 (94.9–110.1)	99.4 (89.8–105.0)	89.1 (79.3–94.6)	81.7 (72.3–87.1)	73.0 (63.3–79.1)	62.5 (54.0–71.4)	91.2 (81.2–101.8)
CKD-EPI-R_Cr-CysC_	120.6 (111.5–127.7)	116.5 (107.4–123.7)	110.8 (101.0–117.9)	101.7 (91.3–110.0)	94.1 (82.9–104.1)	84.2 (72.1–94.7)	74.5 (58.2–86.3)	105.5 (92.9–115.3)
CKD-EPI_CysC_	120.4 (111.4–127.4)	115.9 (108.2–112.5)	110.4 (99.0–116.2)	99.7 (86.7–107.8)	88.7 (75.3–99.6)	75.6 (62.1–87.8)	64.5 (47.3–78.6)	103.7 (88.3–114.2)

Data are presented as median and interquartile range. Abbreviations: CKD, Chronic Kidney Disease; CKD-EPI, Chronic Kidney Disease Epidemiology Collaboration; CKD-EPI-R, Chronic Kidney Disease Epidemiology Collaboration refit; EKFC, The European Kidney Function Consortium; GFR, glomerular filtration rate.

### 3.2. eGFR Based on sCr and sCysC Levels

**Figure 4:**
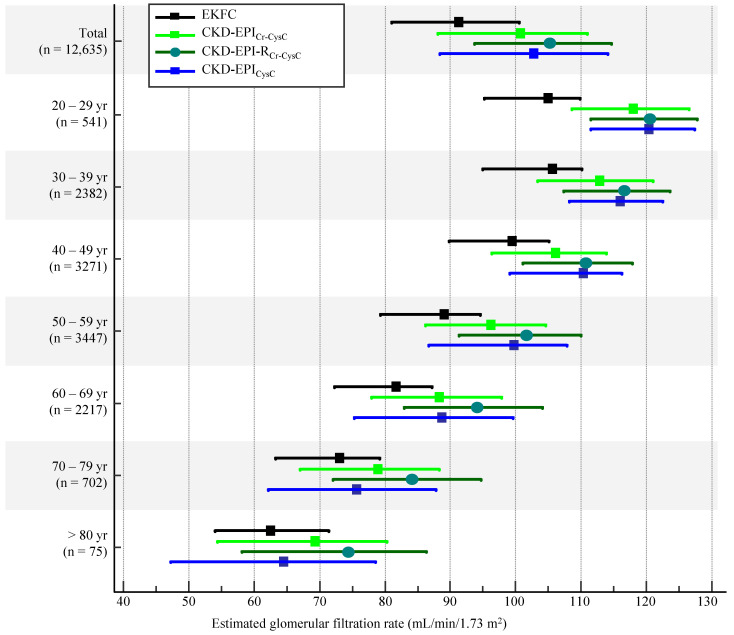
Distribution of eGFR using serum creatinine- and cystatin C-based CKD-EPI, EKFC, and CKD-EPI-R equations stratified by age group. Data are presented as median and interquartile range. Abbreviations: see Table 3.

Similar to the sCr-based equations, the median value of eGFR showed an increasing trend as follows: 91.2 mL/min/1.73 m^2^ using EKFC, 100.4 mL/min/1.73 m^2^ with CKD-EPI_Cr-CysC_, and 105.5 mL/min/1.73 m^2^ with CKD-EPI-R_Cr-CysC_ (Table 3). When the eGFR distribution was stratified by age group, such a trend was consistently observed throughout the entire age group (Figure 4). In the transition age groups of people in their 20s and 30s, the EKFC equation showed the smallest difference of eGFRs between the 20s and 30s, compared with the CKD-EPI_Cr-CysC_ and CKD-EPI-R_Cr-CysC_ equations (0.6 mL/min/1.73 m^2^ vs. 6.2 mL/min/1.73 m^2^ vs. 4.1 mL/min/1.73 m^2^).

Table 4 and Figure 3 show the correlation and difference of eGFRs across CKD-EPI_Cr-CysC_, EKFC, and CKD-EPI-R_Cr-CysC_ equations using the Passing–Bablok regression and the Bland–Altman plot. The CKD-EPI_Cr-CysC_ equation showed a high correlation with the EKFC equation (r = 0.79–0.92) and a very high correlation with the CKD-EPI-R_Cr-CysC_ equation (r = 1.00) in all age groups.

Based on the CKD-EPI_Cr-CysC_ equation, the estimated CKD prevalence was 1.9% (239/12,635) (Table 5). The CKD-EPI-R_Cr-CysC_ equation showed good agreement with the CKD-EPI_Cr-CysC_ equation (κ = 0.81) for potential CKD prevalence; the EKFC equation, however, showed moderate agreement with the CKD-EPI_Cr-CysC_ equation (κ = 0.69). The EKFC equation estimated CKD prevalence as 2.5% (310/12,635); 120 of 12,635 individuals (0.9%) were reclassified as having CKD, while 49 of 12,635 individuals were reclassified as not having CKD. The CKD-EPI-R_Cr-CysC_ equation estimated CKD prevalence as 1.3% (162/12,635); none of the enrolled population was reclassified as having CKD, while 77 of 12,635 individuals (0.6%) were reclassified as not having CKD.

**Table 4:** Correlation and differences of eGFR among serum creatinine- and cystatin C-based CKD-EPI, EKFC, and CKD-EPI-R equations stratified by age group.

Age (yr)	CKD−EPI_Cr−CysC_ vs. EKFC			CKD−EPI_Cr−CysC_ vs. CKD−EPI−R_Cr−CysC_			CKD−EPI−R_Cr−CysC_ vs. EKFC		
	Equation	r (95% CI)	Mean Difference (95% CI)	Equation	r (95% CI)	Mean Difference (95% CI)	Equation	r (95% CI)	Mean Difference (95% CI)
20–29 (n = 541)	y = 0.70x + 20.09	0.79 (0.75–0.82)	14.9 (−2.6–32.4)	y = 0.92x + 11.40	1.00 (1.00–1.00)	−2.0 (−4.6–0.6)	y = 0.76x + 11.10	0.78 (0.74–0.81)	16.9 (0.3–33.5)
30–39 (n = 2382)	y = 0.76x + 17.19	0.81 (0.79–0.82)	9.6 (−6.7–25.9)	y = 0.92x + 11.89	1.00 (1.00–1.00)	−3.1 (−5.6–−0.6)	y = 0.82x + 7.72	0.80 (0.78–0.81)	12.7 (−3.0–28.5)
40–49 (n = 3271)	y = 0.80x + 13.07	0.81 (0.80–0.82)	7.6 (−8.2–23.5)	y = 0.96x + 8.54	1.00 (1.00–1.00)	−4.1 (−6.3–−2.0)	y = 0.84x + 5.68	0.81 (0.80–0.82)	11.8 (−4.1–27.6)
50–59 (n = 3447)	y = 0.76x + 14.12	0.81 (0.79–0.82)	8.3 (−6.6–23.2)	y = 1.01x + 3.73	1.00 (1.00–1.00)	−5.0 (−6.8–−3.1)	y = 0.75x + 11.35	0.78 (0.77–0.80)	13.2 (−2.7–29.1)
60–69 (n = 2217)	y = 0.69x + 18.66	0.83 (0.82–0.84)	8.1 (−7.1–23.3)	y = 1.05x + 1.00	1.00 (1.00–1.00)	−5.3 (−7.4–−3.2)	y = 0.65x + 18.33	0.81 (0.79–0.82)	13.4 (−3.4–30.1)
70–79 (n = 702)	y = 0.69x + 17.28	0.86 (0.84–0.88)	6.8 (−7.7–21.4)	y = 1.07x − 0.37	1.00 (1.00–1.00)	−5.3 (−7.8–−2.8)	y = 0.63x + 17.89	0.84 (0.82–0.86)	12.2 (−4.6–29.0)
>80 (n = 75)	y = 0.65x + 17.55	0.92 (0.88–0.95)	6.0 (−9.4–21.4)	y = 1.09x–0.78	1.00 (1.00–1.00)	−5.2 (−8.4–−1.9)	y = 0.59x + 17.99	0.99 (0.99–0.99)	11.2 (−7.3–29.6)
Total (n = 12,635)	y = 0.84x + 7.41	0.88 (0.88–0.89)	8.5 (−7.3–24.4)	y = 0.96x + 8.27	1.00 (1.00–1.00)	−4.3 (−7.2–−1.5)	y = 0.87x + 0.20	0.87 (0.86–0.87)	12.9 (−3.4–29.1)

All *p* values were <0.001. Equations and correlation coefficients (r) were obtained using Passing–Bablok regression, and mean differences were obtained using Bland–Altman plots. Abbreviations: see Table 1; Cr, creatinine; CysC, cystatin C.

**Table 5:** Agreement of GFR categories among serum creatinine- and cystatin C-based CKD-EPI, EKFC, and CKD-EPI-R equations.

	GFR Category	CKD-EPI_Cr-CysC_							Categorical Agreement		Agreement of Estimated CKD Prevalence			
		G1	G2	G3a	G3b	G4	G5	Total	%	κ (95% CI)	κ (95% CI)	Estimated CKD Prevalence (%)	Difference and Rate in Prevalence (%, 95% CI)	*p*
EKFC	G1	6531	230	0	0	0	0	6761	76.9	0.57 (0.56–0.58)	0.69 (0.64–0.73)	2.5	0.6 (0.2–0.9) 1.3 (1.1–1.5)	0.002
	G2	2500	3015	48	1	0	0	5564						
	G3a	0	120	132	8	0	0	260						
	G3b	0	0	7	32	5	0	44						
	G4	0	0	0	0	4	0	5						
	G5	0	0	0	0	0	1	1						
CKD-EPI-R_Cr-CysC_	G1	9031	1061	0	0	0	0	10,092	90.9	0.78 (0.77–0.79)	0.81 (0.76–0.85)	1.3	−0.6 (−0.9–−0.3) 0.7 (0.6–0.8)	<0.001
	G2	0	2304	77	0	0	0	2381						
	G3a	0	0	110	14	0	0	124						
	G3b	0	0	0	27	2	0	29						
	G4	0	0	0	0	7	1	8						
	G5	0	0	0	0	0	1	1						
CKD-EPI_CysC_	G1	8588	613	0	0	0	0	9201	89.9	0.78 (0.77–0.79)	0.71 (0.67–0.75)	3.1	1.3 (0.8–1.6) 1.7 (1.4–2.0)	<0.001
	G2	443	2584	10	0	0	0	3037						
	G3a	0	167	140	0	0	0	307						
	G3b	0	1	37	39	0	0	77						
	G4	0	0	0	2	9	1	12						
	G5	0	0	0	0	0	1	1						
Total		9031	3365	187	41	9	2	12,635						

Estimated CKD prevalence using the CKD-EPI_Cr-CysC_ equation was 1.9%. Abbreviations: see Table 3.

## 4. Discussion

Current KDIGO guidelines recommend using the CKD-EPI equations in North America, Europe, and Australia; the eGFR equations can be applied in other regions after being evaluated adequately [3,4,39]. This is the first study that has evaluated the newly proposed EKFC and CKD-EPI-R equations in comparison with the current CKD-EPI equations. We compared these equations according to the CLSI guidelines in a large number of the Korean general population and assessed their potential implications in estimating CKD prevalence.

Compared with the CKD-EPI equation, the EKFC equation underestimated GFR, and the CKD-EPI-R equation overestimated GFR (Figure 1 and Table 1). Of note, there was a bigger difference between the EKFC and CKD-EPI-R equations than between the EKFC and CKD-EPI equations. Giavarina D et al. [9] reported similar results, that the EKFC equation underestimated GFR compared with the CKD-EPI equation in an Italian cohort. Inker LA et al. [18] reported that the CKD-EPI equation overestimated mGFR by a median of 0.5 mL/min/1.73 m^2^ and the CKD-EPI-R equation overestimated mGFR by a median of 3.9 mL/min/1.73 m^2^; the mean difference was −3.4 mL/min/1.73 m^2^ in non-Blacks [18]. Recent studies showed that there is a discrepancy between mGFR and eGFR, even in individual levels [40,41]. Although we could not compare eGFR with mGFR directly, the present study also showed the same mean difference (−3.4 mL/min/1.73 m^2^) between CKD-EPI and CKD-EPI-R equations (Table 1). As the National Kidney Foundation (NKF) and American Society of Nephrology (ASN) task force mentioned in their phase 1 interim report, eliminating race from GFR estimation could have clinical consequences; the NKF-ASN task force will develop and review new recommendations through phase 2 and phase 3 [19,42]. It is also noteworthy that the EKFC equation showed the smallest difference in eGFRs between the two age groups of 20s and 30s, compared with the other equations (Figure 2 and Figure 4). This finding was also observed in an Italian population [9]. Taken together, the EKFC equation could be an option to improve GFR estimation in adolescence and young adults, regardless of ethnicity.

In the present study, the eGFR equations showed, statistically, very high correlations and small eGFR differences; however, these equations affected the GFR categories and CKD prevalence significantly. In a French cohort, the CKD prevalence was 1.5% with the CKD-EPI equation and 2.1% with the EKFC equation [12]. Inker LA et al. [18] mentioned that the CKD-EPI-R equation yielded similar or lower CKD prevalence. In our data, compared with the CKD-EPI equation, the EKFC equation reclassified the GFR category downward, while the CKD-EPI-R equation reclassified it upward (Table 2). If the difference in the rate of CKD prevalence in the present study is applied to the national CKD prevalence of the Koreans (9.3%), the EKFC equation would overestimate the CKD prevalence to 13.6%, and the CKD-EPI-R equation would underestimate it to 6.5% [2]. These findings imply that implementing the newly proposed eGFR equations in the Korean population might impact the national CKD prevalence remarkably in an opposite direction; the CKD prevalence using the EKFC equation would be twice as high as that by using the CKD-EPI-R equation. These finding are in line with previous findings reported in other races and ethnicities [9,12,18,39,43,44,45].

The sCysC level is known to be less influenced by ethnicity, and the eGFR using sCysC level is recommended as a confirmatory testing of sCr-based eGFR [18,44]. The CKD-EPI_Cr-CysC_ and CKD-EPI_CysC_ equations are considered more accurate than the CKD-EPI_Cr_ equation for estimating GFR [18,44]. However, in a South Asian cohort with 557 participants, the CKD-EPI_CysC_ equation underestimated mGFR, and the CKD-EPI_Cr-CysC_ equation did not show an advantage compared with the CKD-EPI_Cr_ equation [15]. In the present Korean population, the CKD-EPI-R equations with sCysC levels tended to overestimate GFR compared with the CKD-EPI equations (Figure 4). Compared with the CKD-EPI_Cr-CysC_ equation, the EKFC equation underestimated GFR, and the CKD-EPI-R_Cr-CysC_ equation overestimated GFR (Table 4, Figure 3 and Figure 4). The EKFC and CKD-EPI-R_Cr-CysC_ equations also affected the potential CKD prevalence; if the difference in the rate of CKD prevalence in the present study is applied to the Korean national CKD prevalence (9.3%), the EKFC equation would overestimate the CKD prevalence to 12.1%, and the CKD-EPI-R_Cr-CysC_ equation would underestimate it to 6.3% (Table 5). There has been no study that evaluated the eKFC equation compared with the sCysC-based CKD-EPI or CKD-EPI-R equations. Our findings imply that the transition of eGFR equations would lead to sizable changes in the CKD prevalence regardless of using sCysC level in the equation.

The present study has several limitations. First, we could not compare eGFR with mGFR directly in the study population [3]. Therefore, eGFRs from the equations cannot be interpreted as an overestimation or underestimation of GFR itself. It is well-known that currently used equations overestimate or underestimate GFR [42,46]. Considering that the newly proposed equations also show such limitations, there is room for further modification and optimization of equations to generate more accurate eGFR [18,19,46]. Nevertheless, we aimed to compare the new EKFC and CKD-EPI-R equations with the current CKD-EPI equations, including their potential implications for CKD prevalence; accordingly, mGFR was not imperative to achieve our study purpose. Second, we estimated the potential CKD prevalence based on eGFR only, although CKD is defined based on both GFR and albuminuria according to the KDIGO guidelines [3,21]. Our data, however, are in line with a French cohort, where the CKD prevalence was estimated to be 1.5% with the CKD-EPI equation and 2.1% with the EKFC equation, without the data on albuminuria and/or its duration [12]. Third, this study was based on the Korean general population, and further studies are needed to evaluate the new EKFC and CKD-EPI-R equations in various clinical settings. Fourth, we excluded individuals younger than 20 yrs, and this exclusion criterion was not enough to explore the known benefit of the EKFC equation, which is applicable to the full age spectrum. However, the present study enrolled the largest number of individuals in their 20s (approximately 8000 individuals) compared with previous studies on the EKFC equation [6,7,8,9,10,11,12,13,14,15,16,17]. In addition, we firstly compared the EKFC equation with sCysC-based CKD-EPI and CKD-EPI-R equations in more than 12,000 individuals. Further large-scale studies are also needed to observe the effect of sCysC levels on the eGFR and the potential CKD prevalence. Last, sCr and sCysC levels were measured using the recommended methods with adequate calibration and quality control. However, sCr and sCysC levels can be inevitably affected by analytical and/or biological variations, and such measurement uncertainties could not be eradicated entirely in the present study [47,48,49,50,51].

In conclusion, to our knowledge, this is the first large-scale study that evaluated the newly proposed EKFC and CKD-EPI-R equations compared with the current CKD-EPI equations. The CKD-EPI, EKFC, and CKD-EPI-R equations showed, statistically, very high correlations and good agreements according to the CLSI guidelines. We assessed the potential implication for CKD prevalence in the Korean general population across the equations. Compared with the CKD-EPI equation, the EKFC equation overestimated CKD prevalence and the CKD-EPI-R equation underestimated it. The eGFR equations, based on many uncertain factors, are no one-size-fits-all method to evaluate kidney function, and blindly implementing any equation will lead to a significant change in CKD prevalence. These findings imply that the transition of eGFR equations would lead to notable differences in the potential CKD prevalence. Considering its sizable impact on national kidney health, in-depth discussion is imperative before implementing new eGFR equations in each region.
